# High genetic diversity in *Aegilops tauschii* Coss. accessions from North Iran as revealed by IRAP and REMAP markers

**DOI:** 10.1186/s43141-022-00363-y

**Published:** 2022-06-13

**Authors:** Sona Minaei, Seyyed Abolghasem Mohammadi, Atefeh Sabouri, Ahmad Reza Dadras

**Affiliations:** 1 Department of Plant Breeding and Biotechnology, Faculty of Agriculture, University of Tabriz, Tabriz, Iran; 2 Center of Excellence in Cereal Molecular Breeding, University of Tabriz, Tabriz, Iran; 3 Department of Life Sciences, Center for Cell Pathology, Khazar University, Baku, AZ1096 Azerbaijan; 4 Department of Agronomy and Plant Breeding, Faculty of Agricultural Sciences, University of Guilan, Rasht, Iran; 5 Department of Crop and Horticultural Research, Zanjan Agricultural and Natural Resource Research and Education, AREEO, Zanjan, Iran

**Keywords:** *Aegilops tauschii*, Genetic diversity, IRAP, REMAP, Retrotransposon

## Abstract

**Background:**

*Aegilops tauschii* Coss*.* as a donor of wheat D genome has an important role in wheat breeding programs. Genetic and phylogeographic diversity of 79 *Ae. tauschii* accessions collected from north and northwest of Iran were analyzed based on retroelement insertional polymorphisms using inter-retrotransposon amplified polymorphism (IRAP) and retrotransposon-microsatellite amplified polymorphism (REMAP) markers.

**Results:**

In total, 306 and 151 polymorphic bands were amplified in IRAP and REMAP analyses, respectively. As a result, a high level of polymorphism was observed among the studied accessions as revealed by an average of 25.5 bands per primer/primer combination and mean PIC value of 0.47 in IRAP and an average of 25.16 bands per primer combination and mean PIC value of 0.47 in REMAP. Genetic relationships of the accessions were analyzed using distance- and model-based cluster analyses.

**Conclusion:**

The result showed that genetic distance did not seem to be related to geographic distribution, and the accessions could be divided into three groups, which was further supported by principal coordinate analysis. These results on genetic diversity and population structure of *Ae. tauschii* in Iran should provide important knowledge on genetic resources and their applications in wheat breeding programs.

**Supplementary Information:**

The online version contains supplementary material available at 10.1186/s43141-022-00363-y.

## Background

Approximately, 8000 years ago, a spontaneous hybridization between cultivated emmer wheat (*T. turgidum*; 2n = 4× = 28, AABB) and *Aegilops tauschii* (2n = 2× = 14, DD) in the Fertile Crescent resulted in hexaploid bread wheat (*Triticum aestivum*; 2n = 6× = 42, AABBDD) [1–3]. *Ae. tauschii* Coss. (2n = 2× = 14) as a donor of wheat D genome is a source of many favorable genes for important agronomic traits [4], bread-making quality [5], resistance to Ug99 [6], leaf rust [7], stripe rust [8, 9], grain yield [10], and cadmium (Cd) tolerance [11]. Among the triple genome of the hexaploid wheat and also compared with *Ae. tauschii*, D genome of wheat has the slightest diversity [12]. Therefore, *Ae. tauschii* with the widest geographical distribution and greater genetic variation compared with the corresponding homologous loci in the D genome of bread wheat is a promising genetic resource for broadening wheat genetic background. Considering the wide genetic variation in *Ae. tauschii* germplasm and its easy crossability with wheat, *Ae. tauschii* accessions have been exploited by various groups throughout the world for wheat improvement [13–15].

Based on spike morphology, two subspecies including ssp. *tauschii* with extended cylindrical spikelets and ssp. *strangulata* with moniliform spikes bearing quadrate spikelets have been identified in *Ae. tauschii*. Three distinct varieties, namely var. *anathera*, var. *meyeri*, and var. *typica*, have been recognized within ssp. *tauschii*, while ssp. *strangulat*a contains only the var. *strangulata*. The description of *Ae. tauschii* variants followed the morphological characteristics revealed wide geographical distribution for ssp. *tauschii* around Eurasian continent, while ssp. *strangulata* has a confined dispersal [16]. The ssp. *tauschii* is spread across the entire *Ae. tauschii* geographical range, whereas ssp. *strangulata* is only present in Transcaucasia and around the Caspian Sea region of Iran. However, recently, one ssp. *strangulata* accession is reported from Uzbekistan, which is the first report for this subspecies out of their natural habitat [17]. The analysis of corresponding variants between the D genome of *T. aestivum* and *Ae. tauschii* showed a closer fit with ssp. *strangulata* than ssp. *tauschii*. According to earlier genetic investigations, Transcaucasia and southwestern Caspian Iran are the origins of bread wheat [18, 19]. Wang et al. [3] analyzed the genetic relationships among 477 *Ae. tauschii* and wheat accessions using the *Ae. tauschii* 10 K Infinium single-nucleotide polymorphism (SNP) array and identified 12 *Ae. tauschii* accessions, each closely related to a wheat D genome chromosome. All 12 accessions belonged to spp. *strangulate* collected from southwestern and southern Caspian Sea, Iran. Genetic diversity of *Ae. tauschii’s* germplasm has been extensively studied using various molecular techniques such as allozyme [20], RFLP [19], microsatellite [21–25], IRAP [26], SNPs [3, 27, 28], AFLP [29], and gene sequences [30].

Cereal genomes consisted of an extraordinary number of transposable elements, in particular, LTR retrotransposons, which is highly dynamic. It is reported that the LTR retrotransposons are often found in different densities or copy numbers among individuals of the same species [31–33]. Therefore, several DNA marker techniques have been devised based on LTR retrotransposons which are more common retrotransposon families in plants [32–34]. Owing to generating steady and great insertions in the genome, retrotransposons are known as one of the main creators of genetic diversity and tools for discovering the genomic changes related to their activity [35]. IRAP and REMAP as two retrotransposon-based markers have been widely used to analyze genetic diversity and population structure in various crop plants such as cotton [36], alfalfa [37], barley [38], sunflower [39], and wheat and its wild relatives [40, 41]. IRAP reveals retrotransposon insertional polymorphisms by amplifying the portion of DNA between two retroelements from the same or different families by using a single or paired primer. REMAP reveals polymorphism in the regions amplified between adjacent microsatellite loci and inserted retrotransposon using ISSR and retrotransposon-based primers [42]. IRAP and REMAP markers have been used to assess the genetic diversity and population structure in a collection of 48 Old Portuguese bread wheat cultivars. A high level of polymorphism was revealed by both marker systems; however, the power of IRAP markers was higher in detecting genetic variability at the individual level, but did not differentiate higher taxa. Based on REMAP marker data, botanical varieties were clustered together, and homonym bread wheat cultivars were identified [40]. Taheri et al. [41] assessed the genetic diversity of 14 populations of *T. urartu* and *T. boeoticum* from west and northwest of Iran by IRAP and REMAP markers. Grouping of the accessions using REMAP data could differentiate them according to their species and geographical origin, but grouping based on IRAP could not separate the accessions of two species. However, based on both marker systems, considerable diversity was observed among and within the studied populations.

*Ae. tauschii’s* genome contains at least 66% LTR [43]; therefore, insertional polymorphism could be useful for the detection and evaluation of the level of LTR retrotransposon intraspecific variability in *Ae. tauschii* accessions. Few studies have been utilized retrotransposon-based markers to evaluate genetic variability in collections of *Ae. tauschii*. Saeidi et al. [26] analyzed the genetic relationship of 57 accessions of *Ae. tauschii* from north and center of Iran, using IRAP markers, and reported high levels of genetic diversity in the studied accessions. However, IRAP data could not differentiate the accessions based on their geographical origins. Boyko et al. [44] mapped 80 retrotransposon markers in the high-density and unified cytological and genetic map of *Ae. tauschii*.

In the present study, we used IRAP and REMAP markers to analyze genetic diversity and population structure of a newly collected *Ae. tauschii* accessions from north and northwest of Iran.

## Methods

### Plant materials and DNA extraction

Seventy-nine accessions of *Aegilops tauschii* Coss. were collected from north and northwest of Iran. For each accession, 10–15 plants at least 2 m apart were randomly sampled. The name and geographical information of collection sites were described in the Supplementary table. From each accession, 10–15 seeds were grown in a greenhouse, and DNA was extracted from bulk leaves in a sample of 10–15 plants following the CTAB extraction protocol [45]. DNA quality and quantity were measured using a NanoDrop spectrophotometer at 260 nm and 280 nm wavelengths. The presence of high-molecular-weight DNA was also checked by agarose gel electrophoresis (0.8%).

### IRAP and REMAP analyses

Seven LTR retrotransposon primers from *Sukkula*, *Nikkita*, and *BARE-1* families of barley (*Hordeum vulgare* L.) genome including Sukkula, Nikkita, LTR6150, LTR6149, 5′LTR1, 5′LTR2, and 3′LTR (Table 1) and their combinations were used in IRAP analyses. REMAP analyses were performed using combinations of seven LTR primers used in IRAP and three ISSR primers. The PCRs were performed in a 10-μL reaction mixture containing 4-μL master mix 2× PCR (ready-to-use PCR master mix 2×; Ampliqon), 2 μL DNA template, and 2 μL distilled water. The amplification program consisted of 5 min of initial denaturation at 94 °C followed by 35 cycles of 94 °C for 60 s, annealing at each primer/primer combination specified *T*_*a*_ for 60 s, and 72 °C for 45 s followed with a final extension at 72 °C for 7 min. The amplification products were separated by 4% ultrathin (0.2 mm) non-denature polyacrylamide gel and detected by ethidium bromide staining using a Gel-Scan 3000 electrophoresis system (Corbett, Sydney, Australia).

**Table 1:** Name, retrotransposon source, direction, GenBank accession number, position and sequences of LTR primers, and name and sequences of ISSR primers

Primer	RTN name and orientation	GenBank accession number	Position	Sequence (5′-3′)
***IRAP and REMAP***				
Sukkula	*Sukkula*→	AY054376	4301–4326	GATAGGGTCGCATCTTGGGCGTGAC
Nikkita	*Nikita→*	AY078073 AY078074 AY078075	1–22	CGCATTTGTTCAAGCCTAAACC
LTR6149	*BARE-1→*	Z17327	1993–2012	CTCGCTCGCCCACTACATCAACCGCGTTTATT
LTR6150	*BARE-1←*	Z17327	439–418	CTGGTTCGGCCCATGTCTATGTATCCACACATGTA
3′LTR	*BARE-1→*	Z17327	2112–2138	TGTTTCCCATGCGACGTTCCCCAACA
5′LTR1	*BARE-1←*	Z17327	1–26	TTGCCTCTAGGGCATATTTCCAACA
5′LTR2	*BARE-1←*	Z17327	338–314	ATCATTCCCTCTAGGGCATAATTC
***ISSR***				
ISSR3	(AC) _8_TG			
ISSR6	(ACTG) _4_			
ISSR42	(CA) _7_T			

### Data analysis

The IRAP and REMAP amplification profiles were scored as the presence (1) or absence (0) at each polymorphic band position for each primer/primer combination in all accessions. Each polymorphic band was treated as a single locus with two alleles. Monographic bands were not scored and used in the analyses. The polymorphism information content (PIC), Shannonʼs information index, and Nei’s gene diversity (H_e_) [46] were calculated by GenAlEx 6.503 software [47].

Pairwise, genetic distance between accessions was calculated based on the minimum evolution evolutionary distance coefficient [48] using IRAP and REMAP data, and the correction between two distance matrices was statistically tested by using the mantel test. The ability of IRAP and REMAP markers to reveal genetic relationships among all the *Ae. tauschii* accessions was evaluated by the neighbor-joining algorithm (NJ), for which the trees were constructed using MEGA 7.0 software [49]. Support for the tree was determined by performing 1000 bootstrap operations on the data set generated by distance analysis. Population structure analysis of 79 *Ae. tauschii* accessions was performed based on IRAP and REMAP by using the Bayesian Markov chain Monte Carlo model-based clustering implemented in the software package STRUCTURE v2.3.4 [50]. The model was run by varying the number of subpopulation (K) from 1 to 10 with 5 replications for each K and with a burn-in period of 10,000, followed by 100,000 Markov chain Monte Carlo replications. The optimum number of subpopulation (K) which best estimated the structure of the 79 accessions was predicted based on the log probability of the data [LnP(D)] and delta K (ΔK) [51], using online software STRUCTURE HARVESTER [52]. The accessions were assigned to subpopulations based on their probability of association of ≥ 60% to each of the two groups; accessions with a probability of association < 60% were considered as admixtures. Principal coordinate analysis (PCoA) was performed using GenAlex 6.5 [34] to further investigate the population structure of *Ae. tauschii* accessions. The Nei’ gene diversity (H_e_), Shannon’s information index (I), and within-population variation (WP) were calculated for each geographical population. The analysis of molecular variance (AMOVA) was performed using GenAlex 6.5 [34] to calculate molecular variance components and their statistical significance levels for variation among and within the *Aegilops tauschii* geographical populations.

## Results

### Insertional polymorphism and genetic diversity

#### IRAP

**Fig. 1:**
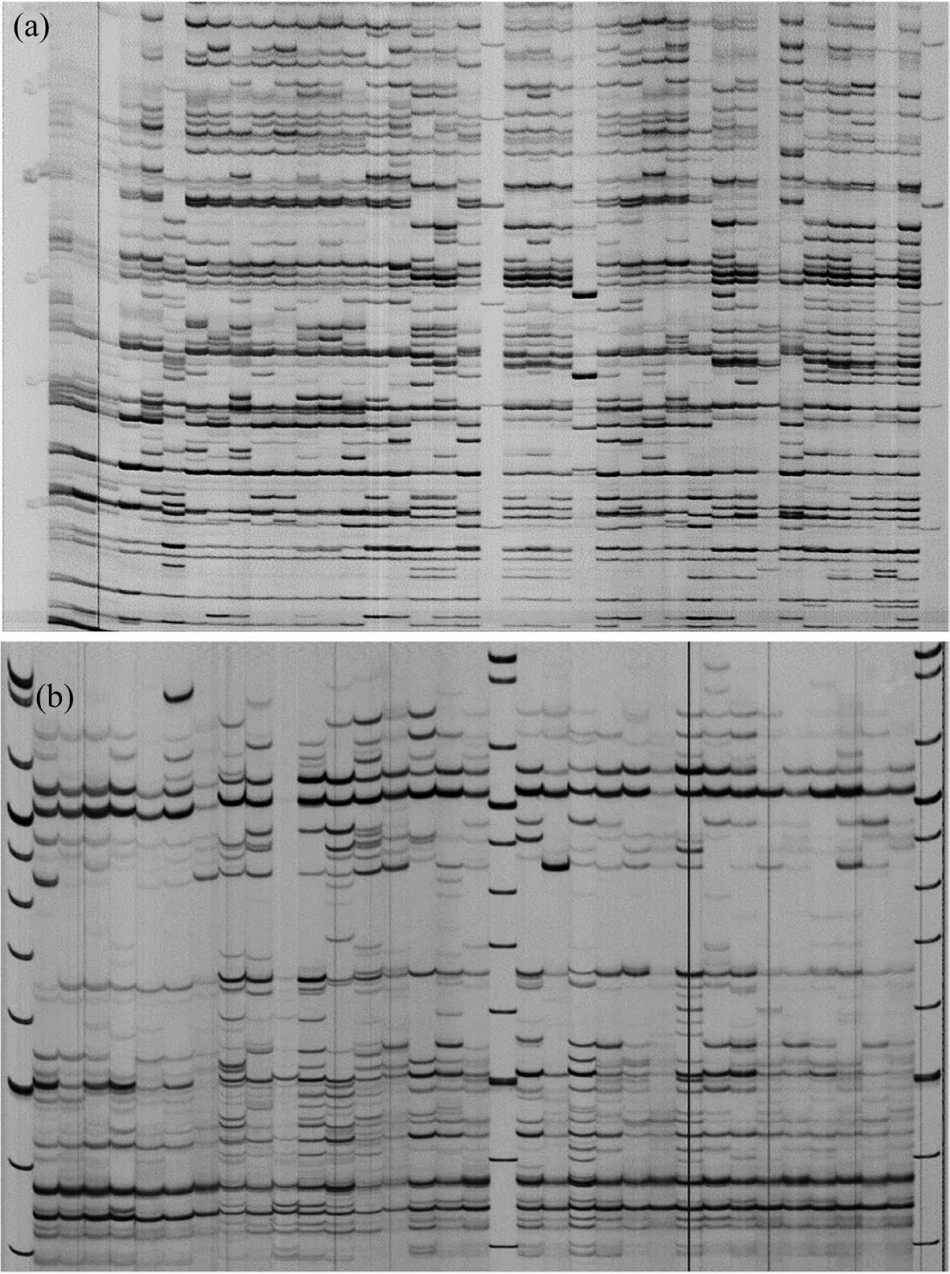
Polyacrylamide gel profile of *Ae. tauschii* accessions using **a** 5′LTR in IRAP and **b** ISSR6 + Sukkula in REMAP

Banding patterns of IRAP amplicons for some *Ae. tauschii* accessions generated using 5′LTR primer are shown in Fig. 1a. In IRAP analysis, out of 28 single primers or primer combinations, five single primers (3′LTR, 5′LTR, Sukkula, Nikkita, and LTR6149) and seven primer combinations produced scorable bands. In total, 306 polymorphic bands were amplified with an average of 25.5 bands per primer/primer combination. The 5′LTR1 primer with 47 and Sukkula primer, and Nikkita + LTR6149 primer combination with 8 bands, produced the highest and lowest number of polymorphic bands in the studied accessions, respectively. The polymorphic information content (PIC) value ranged from 0.36 (LTR6149) to 0.50 (Sukkula + LTR6150) with an average of 0.47. Considering all polymorphic primers/primer combinations, the highest and lowest number of polymorphic loci were amplified in Mazandaran (218) and Azerbaijan (182) populations, respectively. The lowest and highest average number of polymorphic markers were also scored in the populations collected from Azerbaijan (15.17) and Mazandaran (18.17) populations, respectively (Table 2).

**Table 2:** Annealing temperature (Ta), number of polymorphic bands (PB), and polymorphic information content (PIC) for primer/primer combinations in IRAP study

Marker	Ta (°C)	PB				PIC
		Azerbaijan	Golestan	Guilan	Mazandaran	
3′LTR	60	11	15	16	18	0.49
5′LTR1	65	26	24	28	30	0.49
Sukkula	60	6	7	6	7	0.46
Nikkita	58	26	28	28	27	0.49
LTR6149	60	9	8	10	8	0.36
3′LTR +5′LTR1	60	13	16	16	17	0.49
3′LTR + Sukkula	64	11	12	13	16	0.46
3′LTR + LTR6149	60	16	18	18	20	0.48
5'LTR1 + LTR6150	62	26	22	19	35	0.49
Sukkula + LTR6149	64	11	12	12	12	0.48
Sukkula + LTR6150	64	20	22	21	22	0.50
Nikkita + LTR6149	64	7	7	6	6	0.45
Total	-	182	191	193	218	-
Average	-	15.17	15.92	16.08	18.17	0.47

The parameters relating to the genetic diversity of geographical groups including the number of polymorphic loci (NPL), Nei’s gene diversity (He), Shannon’s information index (I), and within-population diversity (WP) are shown in Table 4. The highest (0.40) and lowest (0.24) He values were observed in Guilan and Azerbaijan populations, respectively. Guilan and Azerbaijan populations showed the maximum (0.58) and minimum (0.36) values of Shannon’s index, and the highest and lowest within-population diversity were recorded in Guilan (54.48) and Mazandaran (41.13) populations, respectively.

#### REMAP

Figure 1b shows REMAP banding pattern of some *Ae. tauschii* accessions generated using Sukkula and ISSR6 primers combination. Out of 18 LTR and ISSR primer combinations examined in the REMAP analyses, six primer combinations produced scorable bans. A total of 151 polymorphic bands with an average of 25.16 markers per primer combination were amplified. The highest (44) and lowest (13) number of polymorphic markers were amplified using ISSR6 + 5′LTR1 and ISSR42 + Sukkula combinations, respectively. The PIC value varied from 0.42 (ISSR42 + Sukkula) to 0.49 (ISSR6 + Nikkita and ISSR6 + Sukkula) with an average of 0.47. Based on all polymorphic primer combinations, Mazandaran and Golestan with 88 and 81 loci had the highest and lowest number of polymorphic loci, respectively. The maximum and minimum average number of polymorphic loci were also scored in Mazandaran and Golestan populations with values of 14.67 and 13.50, respectively (Table 3).

**Table 3:** Annealing temperature (Ta), number of polymorphic bands (PB), and polymorphic information content (PIC) for primer/primer combinations in REMAP analysis

Marker	Ta (°C)	PB				PIC
		Azerbaijan	Golestan	Guilan	Mazandaran	
ISSR3 + 5′LTR2	65	12	13	14	19	0.47
ISSR6 + 5′LTR1	64	27	25	26	21	0.48
ISSR6 + Nikkita	65	10	11	11	12	0.49
ISSR6 + Sukkula	64	14	15	15	17	0.49
ISSR42 + 3′LTR	65	13	15	14	15	0.48
ISSR42 + Sukkula	65	9	2	6	4	0.42
Total		85	81	86	88	
Average		14.17	13.50	14.33	14.67	0.47

Based on REMAP data, maximum and minimum values of He were recorded in the Guilan (0.38) and the Mazandaran (0.27) populations, respectively, and Shannon’s index ranged from 0.56 (Guilan) to 0.39 (Mazandaran). The highest level of within-population diversity was observed in the Golestan population (29.18), and the Mazandaran population revealed the lowest within-population diversity (23.40) (Table 2).

Pairwise Nei’s genetic distance among populations was calculated based on IRAP and REMAP data. Based on IRAP data, the highest (0.203) and lowest (0.064) distances were found between “Azerbaijan and Mazandaran” and “Golestan and Guilan,” respectively, which was in agreement with their geographical distances. Among the populations, the Azerbaijan population was far genetically relative to others. In REMAP analysis, the highest genetic distance (0.213) was also observed between Azerbaijan and Mazandaran populations and the lowest (0.043) between Golestan and Guilan populations.

Analysis of molecular variance (AMOVA) revealed high genetic variation within populations and low genetic differentiation among populations. The proportion of variation attributable to within-population differences was high, 90, 94, and 92% by IRAP, REMAP, and combined data, respectively.

### Genetic relationships and population structure

The genetic relationships among 79 *Ae. tauschii* accessions were assessed using the minimum evolution distance coefficient based on IRAP, REMAP, and combined data. Mantel test revealed low and nonsignificant correlation between two matrices (*r* = 0.127, *P* = 0.265). The resulting distance matrices were used to construct dendrograms using the neighbor-joining clustering algorithm. The model-based hierarchical structure among the studied accessions was also conducted by STRUCTURE v2.3.4 software [50] using all three data sets.

**Fig. 2:**
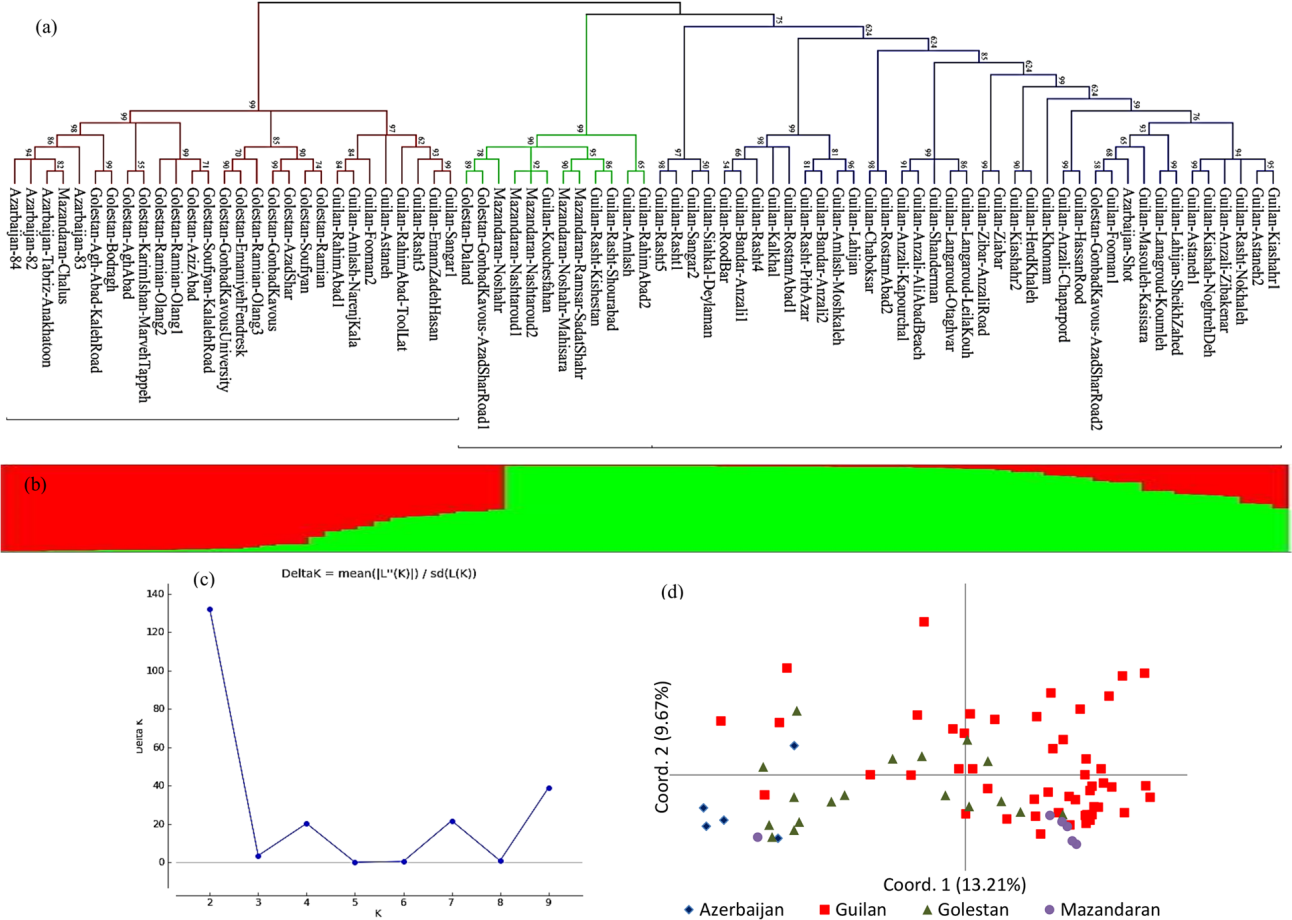
The genetic relationships and population structure of the 79 *Ae. tauschii* accessions based on IRAP data concluded from a distance and model-based cluster analyses and PCoA. **a** Neighbor-joining phylogenetic tree, **b** population structure on *K* = 2, **c** clusters number (*K*) plotted versus Δ*K* for determining ideal *K*-value, and **d** PCoA based on the first two coordinates

Based on IRAP data, the 79 accessions were clustered into 3 groups (Fig. 2a). All the accessions in group 1 belong to Guilan province except one each from Azerbaijan and Golestan provinces. In group 2, most of the rest of Guilan accessions were co-clustered with accessions from Mazandaran and two of Golestan. The majority of accessions from Golestan and Azerbaijan along with some accessions from Guilan and one from Mazandaran constructed group 3. Based on the established phylogenetic lineages using model-based cluster analysis, the relevant population structure was captured at *K* = 2 (Fig. 2b). The 79 *Ae. tauschii* accessions are allocated into two subpopulations which were in agreement with two major groups and which identified using the neighbor-joining tree constructed from the genetic distances among *Ae. tauschii* accessions. The relationships between 79 accessions were also determined using PCoA. The biplot of accessions based on two first coordinates obtained by PCoA led to results comparable to those obtained using distance- and model-based cluster analyses (Fig. 2d). The two first coordinates explained 22.88 of the total IRAP data variation, where coordinates 1 and 2 accounted for 13.21 and 9.67% of the variation, respectively.

**Fig. 3:**
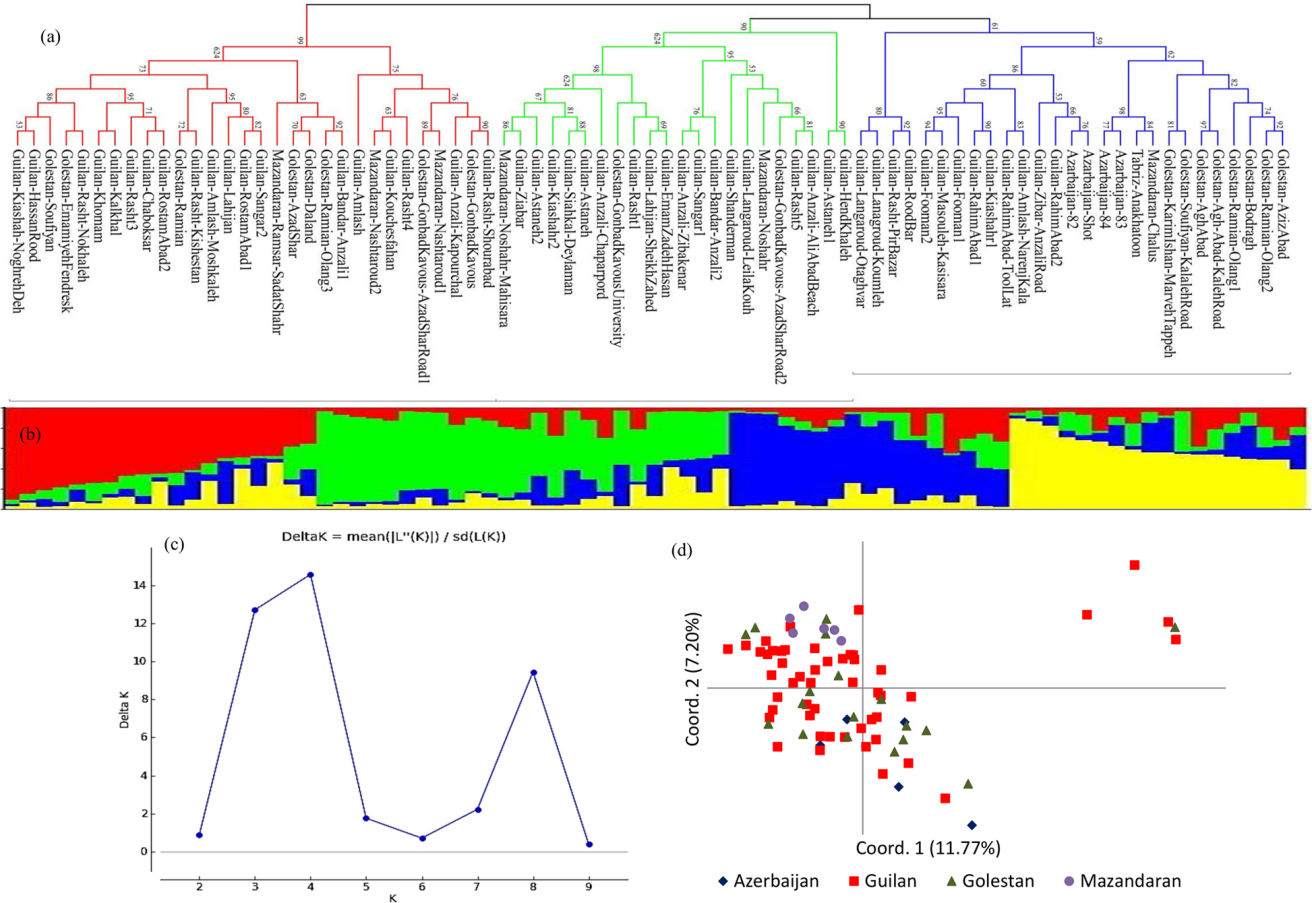
The genetic relationships and population structure of the 79 *Ae. tauschii* accessions based on REMAP data concluded from a distance and model-based cluster analyses and PCoA. **a** Neighbor-joining phylogenetic tree, **b** population structure on *K* = 4, **c** clusters number (*K*) plotted versus Δ*K* for determining ideal *K*-value, and **d** PCoA plot based on the first two coordinates

Distance-based cluster analysis using minimum evolution distance coefficient and N-J algorithm based on REMAP data resulted in a dendrogram assigning the accessions into three distinct groups (Fig. 3a). Group 1 consisted of all accessions from Azerbaijan, the majority of Golestan’s accessions, and some accessions from Guilan. The accessions in group 2 belong to Guilan except two from Mazandaran and two from Golestan. Group 2 was a mixed group including accessions from Guilan, Golestan, and Mazandaran. In the model-based cluster analysis, the maximum value for Δ*K* was observed when *K* = 4 (Fig. 3c), indicating the presence of four main population groups (Fig. 3b). The result of model-based cluster analysis does not fully support the grouping obtained by the N-J distance-based clustering. The distribution of the 79 *Ae. tauschii* accessions on the basis of REMAP data was explained by the first two principal coordinates, where the first and second coordinates explained 11.77 and 7.20% of the total variation among the accessions, respectively, and could not show a clear separation of accessions from different provinces, although the accessions from the single province were grouped closely (Fig. 3d).

**Fig. 4:**
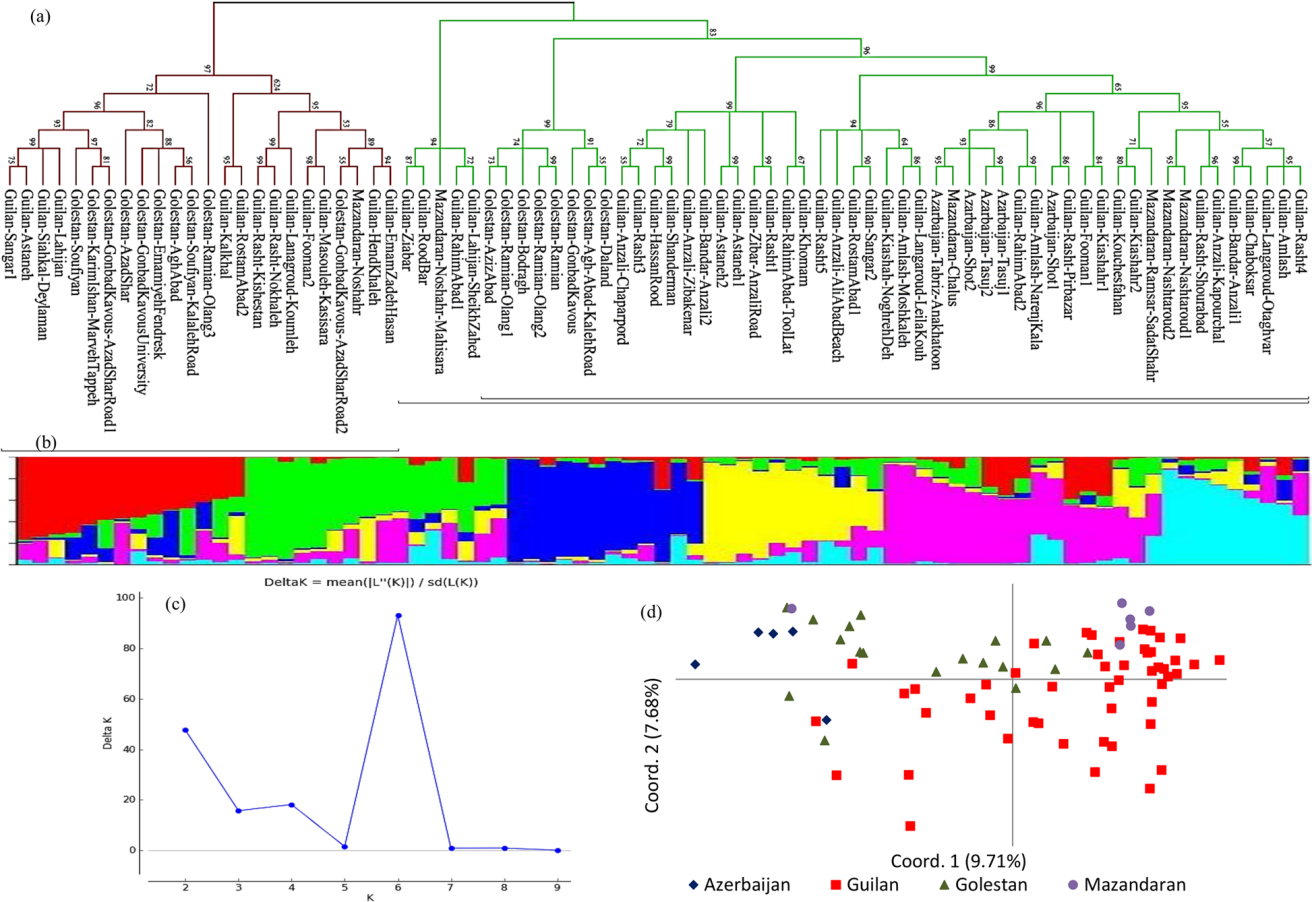
The genetic relationships and population structure of the 79 *Ae. tauschii* accessions based on IRAP + REMAP data concluded from a distance and model-based cluster analyses and PCoA. **a** Neighbor-joining phylogenetic tree, **b** population structure on *K* = 6, **c** clusters number (*K*) plotted versus Δ*K* for determining ideal *K*-value, and **d** PCoA plot based on the first two coordinates

Cluster analysis using the N-J algorithm based on combined IRAP and REMAP could not clearly separate accessions from different provinces. In the resulting phylogenetic tree, the accessions were assigned into two groups. Group 1 was a mixed group that consisted of accessions from the four provinces including all accessions of Azerbaijan and Mazandaran and the majority of Guilan’s accessions along with some from Colestan. In group 2, the rest accessions of Guilan and Golestan were co-clustered (Fig. 4a). In model-based clustering, to find out the ideal *K*-value, the number of clusters (*K*) was plotted versus Δ*K*, which presented a clear peak at *K* = 6 (Fig. 4c). With six groups, the majority of the accessions collected from Guilan province were clustered together, and Golestan’s accessions were also clustered closely (Fig. 4b). In PCoA using combined data, the first and second coordinates accounted for 5.82 and 4.60% of the total variation, respectively. Biplot derived from the PCoA of the 79 *Ae. tauschii* accessions could not display clear relationships among accessions according to their collected provinces, and the accessions from the same province did not closely group (Fig. 4d). However, the results of PCoA agreed with STRUCTURE analyses compared with N-J clustering.

## Discussion

Various DNA markers including AFLP [53], SSR [24], retrotransposon-based markers [26, 53], DArT [54], and SNP [28] have been extensively used to analyze the genetic diversity and population structure of *Ae. tauschii* collections. Among the various DNA fragments used as DNA markers, the transposable elements have been reported as drivers of structural and functional variations in the wheat genome. Transposable elements are major contributors to genome plasticity and thus are likely to have a dramatic impact on genetic diversity and speciation [55].

In the present study, the genetic diversity and population structure of 79 *Ae. tauschii* from north and northwest of Iran were assessed by insertional polymorphism using two retrotransposon-based markers: IRAP and REMAP. As a result, a high level of polymorphism was observed among the studied accessions as revealed by an average of 25.5 bands per primer/primer combination and mean PIC value of 0.47 in IRAP and an average of 25.16 bands per primer combination and a mean PIC value of 0.47 in REMAP analyses. Safiyar et al. [56] used 15 IRAP and REMAP markers to analyze the genetic diversity of *Ae. tauschii* collections from different regions of Iran. They reported a high level of polymorphism for both markers, but the level of polymorphism was higher for IRAP compared with REMAP as detected by the effective number of alleles, Nei’s gene diversity, and Shannon’s information index. Saeidi et al. [26] detected a high level of genetic diversity among 57 *Ae. tauschii* accessions from northern and Central Iran by means of retroelement insertional polymorphisms generated using IRAP markers. The REMAP markers were used for molecular diversity analysis of 45 genotypes from four *Aegilops* species, and a high number of polymorphic bands (96.09% of polymorphism) with a mean PIC value of 0.25 was produced [57].

Retrotransposons as major generators of genetic diversity and tools for detecting the genomic changes created by their large and stable insertions in the genome have been widely applied in plant genetic studies [34, 58]. The IRAP and REMAP methods are two common retrotransposon-based markers that were first implemented in *barley* for fingerprinting and biodiversity studies [50, 59]. Due to the evidence that the closely related retrotransposons are transcribed and translated in diverse grass species belonging to different subfamilies [60], we utilized seven LTR retrotransposon primers developed from *Sukkula*, *Nikkita*, and *BARE-1* families of barley to analyze genetic relationships among *Ae. tauschii* accessions. Five out of the seven examined primers and seven primer combinations allowed the amplification of multiple and distinguishable fragments in the 79 *Ae. tauschii* accessions, and the degree of polymorphism was very high, 100% by either method. In addition to the detection of high IRAP and REMAP polymorphism among the 79 accessions, polymorphism within each geographical population was also high for most of the primers/primer combinations (Tables 2 and 3). In our study, the inability of some primers/primer combinations to amplify fragments from *Aegilops* genome may be the limited dispersal of the barley’s retrotransposon families that were used to design the primers in the *Ae. tauschii* genome.

The values of intrapopulation genetic diversity based on Nei’s gene diversity using IRAP data ranged from 0.24 among accessions from the Azerbaijan population to 0.48 among accessions from the Guilan population. Based on REMAP data, it ranged from 0.27 for Mazandaran’s accessions to 0.38 for Guilan’s accessions. The same trends within-population genetic diversity were observed based on Shannon’s information index (Table 4). The higher and lower within-population diversity could be due to the number of accessions sampled from each population. Singh et al. [17] used SNP markers to compute Nei’s diversity index for *Ae. tauschii* lineage 1 (L1), lineage 2 (L2), possible hybrids, wheat, and *Ae. tauschii* collection combined and reported the highest Nei’s diversity index for L2 = 0.1326 followed by L1 = 0.0872 and wheat of 0.0158. Higher values of the Nei’s index indicate greater allelic diversity in a given population.

**Table 4:** Nei’s gene diversity (H_e_), Shannon’s information index of genetic diversity (I), and within-population variation (WP) of geographical populations based on IRAP and REMAP data

Population	IRAP			REMAP		
	H_**e**_	I	WP	H_**e**_	I	WP
**Azerbaijan**	0.24	0.36	47.80	0.29	0.42	27.60
**Golestan**	0.36	0.52	51.95	0.37	0.55	29.18
**Guilan**	0.40	0.58	54.48	0.38	0.56	27.69
**Mazandaran**	0.25	0.37	41.13	0.27	0.39	23.40
**Mean**	0.31	0.46		0.33	0.48	

The phylogenetic trees were constructed by two different clustering methods to analyze the genetic relationships of 79 *Ae. tauschii accessions* using IRAP, and REMAP data did not agree fairly well with the accession’s provinces. However, in most cases, the accessions from the same provinces were closely clustered (Figs. 2, 3, and 4). This could be due to the fact that the accessions from a province have been collected from locations with different geographical and ecological properties. In general, there was no clear relationship between the genetic distance calculated using marker data and the geographical distance for the populations, which may result from the high degree of polymorphism found within the populations. Closer genetic relationships among accessions which geographically located distantly may likely implicate long-distance seed distribution. Analysis of genetic relationships among 57 accessions of *Ae. tauschii* from northern, northwest, northeast, and Central Iran using retroelement insertional polymorphisms generated by the IRAP method revealed that the accessions from the northwest and central were co-clustered with the accessions from north and northeast, and no clear grouping was observed based on the geographical origins [26]. It is reported that the amplification of transposable elements particularly retroelements is under major environmental effect [61–63]; therefore, the conditions in the regions favor activity and dispersion of the elements.

Mantel test revealed a low and nonsignificant correlation between the two matrices (*r* = 0.127, *P* = 0.265). It could be due to different genomic regions amplified by these two marker systems. Using the IRAP, genomic regions are amplified by two nearby retrotransposons using outward-facing primers, whereas, in REMAP, amplification between retrotransposons proximal to simple sequence repeats (microsatellites) produces the marker bands (59, 60).

## Conclusion

Wang et al. [3] used 7815 SNPs providing complete coverage of the genome to interrogate 402 accessions of *Ae. tauschii*, 75 hexaploid wheats, and seven tetraploid wheats conclude that southwestern Caspian Iran is the center of wheat genetic diversity and the center of origin of bread wheat. Therefore, studying *Ae. tauschii* accessions from the wheat center of origin using molecular markers has an essential role in wheat breeding programs. We analyze the genetic diversity of 79 accessions mainly collected from the Caspian area using two highly polymorphic markers: IRAP and REMAP. The results of the molecular genetic diversity analysis clearly showed that this collection of *Ae. tauschii* will be helpful for wheat breeding programs as revealed by high PIC and diversity indices and the number of polymorphic markers.

## Supplementary Information

**Additional file 1: Supplementary table.** Name and geographical coordination of the studied *Ae. tauschii* accessions.

## Data Availability

All data generated or analyzed during this study are included in this published article.

## Abbreviations

Ae: *Aegilops*
T: *Triticum*
IRAP: Inter-retrotransposon amplified polymorphism
REMAP: Retrotransposon-microsatellite amplified polymorphism
RFLP: Restriction fragment length polymorphism
AFLP: Amplified fragment length polymorphism
SNP: Single-nucleotide polymorphism
DArT: Diversity arrays technology
SSR: Simple sequence repeat
ISSR: Inter-simple sequence repeats
PIC: Polymorphism information content
He: Nei’s gene diversity
I: Shannon information index
PB: Number of polymorphic bands
NJ: Neighbor joining
PCoA: Principal coordinate analysis
