# Supplementary material for: High genetic diversity in Aegilops tauschii Coss. accessions from North Iran as revealed by IRAP and REMAP markers
Source: J Genet Eng Biotechnol. 2022 Jun 13;20:86. doi: 10.1186/s43141-022-00363-y (PMC9192835; doi:10.1186/s43141-022-00363-y)
Supplement: Supplementary file 1 — Additional file 1: Supplementary table. Name and geographical coordination of the studied Ae. tauschii accessions. [file 43141_2022_363_MOESM1_ESM.docx]

**Supplementary Table**. Name and geographical coordination of the studied *Ae. tauschii* accessions

| Collection | E | N | A | Tmax | Tmin |
| --- | --- | --- | --- | --- | --- |
| Guilan-Lahijan | 49° 59' 33.75" | 37° 12' 39.23" | 2.13 | 20.1 | 11 |
| Guilan-Astaneh | 49° 56' 57.7" | 37° 15' 51.7" | -6.75 | 19.1 | 11 |
| Guilan-Sangar | 49° 39' 43.6" | 37° 11' 53" | 29.48 | 20.6 | 11.5 |
| Guilan-Emam Zadeh Hasan | 49° 37' 28" | 37° 1' 21.8" | 2.13 | 21 | 12 |
| Guilan-Siahkal,Deylaman | 49° 54' 36.1" | 36° 53' 27.8" | 1429.6 | 20.1 | 11 |
| Guilan-Sangar | 49° 41' 0" | 37° 11' 0" | 29.48 | 20.6 | 11.5 |
| Guilan-Rostam Abad | 49° 30' 54.7" | 36° 55' 46" | 155 | 20.5 | 10 |
| Guilan-Rostam Abad | 40° 30' 5.2" | 36° 55' 4.9" | 155 | 20.5 | 10 |
| Guilan- Chaboksar | 50° 34' 0" | 36° 57' 0" | -9.83 | 21 | 11.5 |
| Guilan-Rasht | 49° 35' 36.52" | 37° 15' 41.79" | 2.55 | 20.6 | 11.5 |
| Guilan-Bandar-Anzali | 49° 22' 3" | 37° 29' 8" | -23 | 20.6 | 11 |
| Guilan-Bandar-Anzali | 49° 27' 44" | 37° 28' 22" | -23 | 20.6 | 11 |
| Guilan-Khalkhal | 48° 56' 38.7" | 37° 42' 11.1" | 1948 | 18 | 7 |
| Guilan-Shanderman | 49° 0' 32.2" | 37° 43' 15.3" | 64 | 19 | 10 |
| Guilan-Rasht | 49° 39' 37.34" | 37° 10' 42.7" | 2.55 | 20.6 | 11.5 |
| Guilan-Rasht | 49° 39' 4.43" | 37° 5' 10.85" | 2.55 | 20.6 | 11.5 |
| Guilan-Roodbar | 49° 32' 52.5" | 36° 58' 1.24" | 188.98 | 22 | 12 |
| Guilan-Rasht,Pirbazar | 49° 30' 6.88" | 37° 20' 31.1" | 2.55 | 20.6 | 11.5 |
| Guilan-Rasht,Nokhaleh Village | 49° 28' 51.56" | 37° 21' 31.85" | 2.55 | 20.6 | 11.5 |
| Guilan-Hend Khaleh | 49° 26' 39.76" | 37° 21' 41.37" | -25 | 20.6 | 11 |
| Guilan-Ziabar | 49° 16' 33.39" | 37° 25' 12.7" | 2.06 | 19 | 10 |
| Guilan-Zibar,Anzali Road | 49° 18' 39.28" | 37° 28' 16.61" | 2.06 | 20.6 | 11 |
| Guilan-bandar-Anzali,Ali Abad Beach | 49° 14' 47.38" | 37° 32' 44.02" | -23 | 20.6 | 11 |
| Guilan-bandar-Anzali,Kapourchal | 49° 21' 16.67" | 37° 30' 10.69" | -23 | 20.6 | 11 |
| Guilan-Khomam | 49° 38' 42.5" | 37° 22' 19.9" | -16.11 | 21 | 12 |
| Guilan-Hassan Rood | 49° 37' 15.69" | 37° 27' 20.68" | -24 | 20.6 | 11 |
| Guilan-Chaparpord,Anzali | 49° 42' 56.98" | 37° 26' 17.62" | -23 | 20.6 | 11 |
| Guilan-Zibakenar,Anzali | 49° 49' 42.78" | 37° 26' 14.5" | -23 | 20.5 | 11 |
| Guilan-Kiashahr | 49° 56' 24.73" | 37° 25' 9.9" | -22.24 | 20.6 | 11.5 |
| Guilan-Kiashahr,Noghreh deh village | 49° 55' 56.59" | 37° 22' 12.34" | -22.24 | 20.6 | 11.5 |
| Guilan-Astaneh | 49° 57' 5.75" | 37° 17' 31.95" | -6.75 | 19.1 | 11 |
| Guilan-Astaneh | 49° 53' 19.98" | 37° 16' 3.62" | -6.75 | 19.1 | 11 |
| Guilan-Kiashahr | 49° 59' 35.72" | 37° 24' 41.69" | -22.24 | 20.6 | 11 |
| Guilan-Fooman | 49° 17' 44.19" | 37° 13' 19.22" | 34 | 21 | 10 |
| Guilan-Masouleh,Kasisara Village | 49° 10' 28.37" | 37° 11' 0.5" | 1289.22 | 21 | 9 |
| Guilan-Fooman | 49° 18' 55.51" | 37° 14' 1.64" | 34 | 21 | 10 |
| Guilan-Rasht | 49° 34' 50.38" | 37° 13' 30.46" | 2.55 | 20.6 | 11.5 |
| Guilan-Lahijan,Sheikh Zahed | 50° 2' 23.45" | 37° 12' 10.82" | 2.13 | 20.1 | 11 |
| Guilan-Langaroud,Leila Kouh | 50° 8' 19.25" | 37° 10' 44.93" | -17.44 | 20.1 | 11 |
| Guilan-Koumleh,Lanagroud | 50° 10' 9" | 37° 8' 24.42" | -17.44 | 20.1 | 11 |
| Guilan-Otaghvar,Langaroud | 50° 6' 53.86" | 37° 6' 47.16" | -17.44 | 20.1 | 11 |
| Guilan-Amlash,Moshkaleh Vilage | 50° 11' 47.21" | 37° 4' 38.16" | 36.32 | 20.1 | 11 |
| Guilan-Amlash,Narenj Kala | 50° 18' 7.06" | 37° 2' 19.72" | 36.32 | 20.1 | 11 |
| Guilan-Rahim Abad,Tool Lat | 50° 17' 17.09" | 36° 59' 32.56" | 47 | 20.5 | 11 |
| Guilan-Rahim Abad | 50° 19' 44.55" | 37° 1' 41.07" | 47 | 20.5 | 11 |
| Guilan-Rahim Abad | 50° 20' 44.96" | 37° 0' 22.88" | 47 | 20.5 | 11 |
| Guilan-Amlash | 50° 11' 51.8" | 37° 5' 34.2" | 36.32 | 20.1 | 11 |
| West Azerbaijan- Showt | 44° 44' 698" | 39° 13' 198" | 1019.56 | 17.8 | 5.4 |
| Guilan-Rasht,Shourabad | 49° 29' 53.37" | 37° 20' 33.21" | 2.55 | 20.6 | 11.5 |
| Guilan-Rasht,Kishestan | 49° 29' 11.75" | 37° 20' 4.4" | 2.55 | 20.6 | 11.5 |
| Guilan-Kouchesfahan | 49° 46' 7.76" | 37° 17' 1.93" | 2.55 | 20.6 | 11.5 |
| Mazandaran-Sadat Shahr,Ramsar | 50° 41' 22.9" | 36° 53' 7.7" | 9.02 | 22 | 12 |
| Mazandaran-Noshahr,Mahisara Beach | 51° 30' 57.6" | 36° 39' 6.2" | -16 | 22.5 | 12 |
| Mazandaran-Nashtaroud | 51° 0' 32.7" | 36° 45' 28" | -22.17 | 22.5 | 13.3 |
| Mazandaran-Nashtaroud | 51° 0' 33.1" | 36° 45' 29.1" | -22.17 | 22.5 | 13.3 |
| Mazandaran-Noshahr | 51° 28' 42.4" | 37° 39' 46.6" | -16 | 22.5 | 12 |
| Golestan-Gonbad Kavous, Azad Shar Road | 55° 9' 52.55'' | 37° 12' 56.96'' | 40 | 23 | 12.7 |
| Golestan-Daland | 55° 2' 47.69'' | 37° 2' 6.66'' | 56 | 24 | 12 |
| Golestan-Soufiyan,Kalaleh Road | 55° 29' 46.3" | 37° 24' 24.1" | 155.54 | 23 | 12.5 |
| Golestan-Karim Ishan,Marveh Tappeh,Aziz Abad Road | 55° 45' 46.1" | 37° 41' 53.7" | 594 | 22 | 11 |
| Golestan-Ramian | 55° 8' 25.23'' | 37° 0' 52.75'' | 225.92 | 24 | 12 |
| Golestan-15 Km Ramian,Olang | 55° 37' 19.67'' | 37° 8' 58.20'' | -4.2 | 24 | 13 |
| Golestan-Aziz Abad | 55° 37' 45.1" | 37° 34' 48.2" | 289 | 22 | 11 |
| Golestan-Ramian,Olang | 55° 37' 19.67'' | 37° 8' 58.20'' | -4.2 | 24 | 13 |
| Golestan-Gonbad Kavous, Azad Shar Road | 55° 9' 52.55'' | 37° 12' 56.96'' | 40 | 23 | 12.7 |
| Golestan-Ramian,Olang | 55° 37' 19.67'' | 37° 8' 58.20'' | 225.92 | 24 | 12 |
| Golestan-AzadShar | 55° 10' 14.52'' | 37° 5' 2.03'' | 127 | 23 | 12.7 |
| Golestan-Gonbad Kavous | 55° 9' 34.77'' | 37° 14' 26.40'' | 40 | 23 | 12.7 |
| Golestan-Emamiyeh Fendresk | 54° 57' 27.87'' | 37° 0' 12.64'' | 225.92 | 24 | 12 |
| Golestan-Gonbad Kavous University | 55° 11' 10.42'' | 37° 15' 46.20'' | 40 | 23 | 12.7 |
| Golestan-Bodragh | 54° 49' 20.11'' | 37° 4' 51.39'' | 36.07 | 23 | 12.5 |
| Golestan-Agh Abad,Kaleh Road | 55° 23' 2.4" | 37° 18' 11.9" | 155.54 | 23 | 12.5 |
| Golestan-Agh Abad | 55° 14' 40.3" | 37° 16' 39.6" | 51 | 23 | 12.5 |
| Golestan-Soufiyan | 55° 30' 42.4" | 37° 29' 4" | 164 | 23 | 12.5 |
| Mazandaran-Chalous | 51° 18' 58.5" | 36° 26' 6.576" | 80.55 | 22.5 | 13.3 |
| Tabriz- Anakhatoun | 46° 14' 40.13'' | 38° 7' 24.06'' | 1406 | 18.2 | 7 |
| West Azerbaijan- Showt | 44° 45' 273" | 39° 14' 799" | 1019.56 | 17.8 | 5.4 |
| East Azaerbaijan- Tasuj | 45° 29' 273" | 38° 27' 706" | 1365 | 18.5 | 6.5 |
| East Azaerbaijan- Tasuj | 45° 29' 373" | 38° 27' 706" | 1365 | 18.5 | 6.5 |
